# The Temperature Influence on Drying Kinetics and Physico-Chemical Properties of Pomegranate Peels and Seeds

**DOI:** 10.3390/foods12020286

**Published:** 2023-01-07

**Authors:** Roberta de Oliveira Sousa Wanderley, Rossana Maria Feitosa de Figueirêdo, Alexandre José de Melo Queiroz, Francislaine Suelia dos Santos, Yaroslávia Ferreira Paiva, João Paulo de Lima Ferreira, Antônio Gilson Barbosa de Lima, Josivanda Palmeira Gomes, Caciana Cavalcanti Costa, Wilton Pereira da Silva, Dyego da Costa Santos, Patricio Borges Maracajá

**Affiliations:** 1 Department of Process Engineering, Federal University of Campina Grande, Campina Grande 58429-900, Brazil; 2 Department of Agricultural Engineering, Federal University of Campina Grande, Campina Grande 58429-900, Brazil; 3 Department of Technology in Agroindustry, Federal Institute of Education, Scienceand Technology of RioGrande do Norte, Paus dos Ferros 59900-000, Brazil; 4 Animal Production Core, National Semiarid Institute, Campina Grande 58434-700, Brazil

**Keywords:** *Punica Granatum* L., effective diffusivity, thermodynamic properties

## Abstract

Pomegranate is a fruit desirable for its nutritional and medicinal properties which has a great industrial potential that is yet under-explored. Notable for its integral use, the peels are used in medicinal infusions and the seeds consumed without restrictions. In this sense, the objective of this work is to determine the drying kinetics of pomegranate peels and seeds in a hot air circulation oven, at temperatures of 50, 60, and 70 °C, adjust mathematical models to experimental data, determine the effective diffusivities and thermodynamic properties of the process and the physicochemical characteristics of peels and seeds of fresh pomegranates and in their flours. Twelve models were used to adjust the drying kinetics, obtaining better results with the Diffusion Approximation model, Verma, and modified Henderson and Pabis. The effective diffusivities were well represented by an Arrhenius equation, with activation energies of 31.39 kJ/mol for seeds and 10.60 kJ/mol for peels. In the drying process, the seeds showed higher values of enthalpy, entropy, and Gibbs free energy concerning peels. Pomegranate peel and seed flours have proximal composition and distinct physicochemical characteristics, with high fiber, carbohydrate, and energy content. In addition, peel flours stand out for their mineral content, and seed flours do for their lipid and protein content.

## 1. Introduction

The pomegranate tree (*Punica granatum* L.) a plant of the Punicaceae family which produces a traditionally consumed fruit that has medicinal effects. The pomegranate is a spherical berry with a thick, reddish-brown or yellow skin and many seeds. Delimited by a pericarp, incorporating numerous arils, denomination is assigned to the fleshy covering of the seeds, and each seed is surrounded by a translucent sac containing the reddish juice [1]. Thus, the fruit itself produces three parts: the seeds, which correspond to approximately 3% of the weight of the fruit and consist of approximately 20% oil; the juice, representing about 30% of the weight of the fruit; and the shells (pericarp), which include the inner network of membranes and account for 67% of the total weight [2].

Pomegranate cultivation dates back to the Middle East, spreading through arid and semi-arid regions, including Brazil, especially in the Northeast [3]. The main pomegranate-growing and producing regions are Iran, Afghanistan, India, Mediterranean countries such as Morocco, Spain, Turkey, Tunisia, Egypt, and Middle Eastern countries [4,5]. Its possibilities for expansion in arid and semi-arid areas of the world are enormous, especially where salinity and water scarcity are limiting factors for other cultures. Its consumption has been reported since antiquity in biblical and mythological texts, being linked to fecundity, wealth, and medicinal treatment of different pathologies [6]. The pomegranate is a plant of multiple uses, with people taking advantage of the peel and pulp, as well as flowers, leaves, and bark of the branches, reporting that the extracts of these parts have antioxidant, antifungal, and antibacterial properties, and can also be used in the fight against inflammation and proliferation of cancer cells [7,8].

In the literature, there are indications of the effect of pomegranate against several diseases, such as cancer, type 2 diabetes, atherosclerosis, and cardiovascular diseases using all the fruit and its derivatives, such as seed oil, bark, extract of flowers, and the juice [9].

Pomegranate can be used as a natural additive in food preservation, leading to the increasing use of its compounds by the food industry [10]. Pomegranate has gained popularity due to its multifunctionality and nutritional value, however, despite this, the low shelf life of fresh fruits compromises consumption. Among the methods to prolong the useful life of fruits and their derivatives, dehydration is one of the oldest and most frequently used options [11].

The most accurate way of studying the behavior of agricultural products in drying processes is to experimentally determine the kinetics of water loss and adjust them using models available in the literature whose predictive capacity is among the highest. The adjustment of mathematical models to experimental drying data, according to the characteristics of each species, is of great importance in decision-making and contributes to improving the efficiency of the drying process [12].

Given the above, despite the large amount of waste generated in the processing of pomegranates, they have appreciable nutritional and sensory characteristics, containing compounds with high biological activity. Taking into account that pomegranate use can improve the performance of the agroindustry, minimize the generation of waste, add value to by-products, there is a consequent reduction of the environmental impact associated with the activity [13]. This work aimed to evaluate the drying kinetics of pomegranate peels and seeds at different temperatures (50, 60, and 70 °C), adjust mathematical models to experimental data, determine effective diffusion and thermodynamic properties of the process and characterize the materials into fresh and dehydrated in the form of flour, examining their proximal composition and physicochemical characteristics.

## 2. Materials and Methods

### 2.1. Raw Materials and Processing

Mature (completely red bark color) pomegranates were used. The fruits were washed in running water and sanitized with the 200 ppm chlorinated solution; then, they were manually peeled; the seeds were withdrawn with pulp from the shell, then the seeds with the pulp were placed in an industrial depulper (Laboremus, Campina Grande, PB, Brazil) to remove the pulp; the peels and seeds were separately packed in plastic bags and stored under freezing temperatures for use in the following stages of the work.

### 2.2. Drying

Initially, the water content of the samples was determined by the drying greenhouse method, at 105 ± 3 °C, for 24 h [14], which presented 70.74% for the bark and 36.60% for the seeds, respectively. For drying, the pomegranate peels were cut longitudinally to 7 cm in length and 1 cm in width, distributed in stainless steel trays and these dimensions subjected to drying; the seeds were distributed in thin layers of stainless steel trays, also subjected to drying under the same conditions as the peel. 

The drying of the samples (peels and seeds), in triplicate, was performed in an oven with forced air circulation (Fanem, model 320, Guarulhos, São Paulo, Brazil) at temperatures of 50, 60, and 70 °C and air velocity of 1.0 m s^−1^ until the equilibrium water content was reached. The reduction of water content during drying was monitored by the gravimetric method (mass loss), weighing the samples at time intervals of 5, 10, 15, 20, 30, 40, 50, and 60 min, using a semi-analytical balance (Marte, model AS5500C, Santa Rita do Sapucaí, Minas Gerais, Brazil). 

With the water content data, the values of the water content ratio were calculated according to Equation (1):

(1) $$\mathrm{MR}=\frac{X\;-{\;X}_{e}}{X_{0\;}-{\;X}_{e}}$$

where MR—Product water content ratio (dimensionless); X—Actual product water content (b. s.); X_0_—Initial water content of the product (b. s.); X_e_—Product balance water content (b. s.).

#### 2.2.1. Drying Kinetic Adjustment Models

Mathematical models allow the simulation of the results obtained in an experimental field to use them in the scale of industrial processes, aiming to predict adequate drying conditions, process performance, dryers, or drying systems [15]. Then, to describe the drying kinetics of pomegranate peels and seeds, the mathematical models presented in Table 1 were used.

To assess the quality of the models that fit the experimental data, the coefficient of determination (R^2^) (Equation (14)), the mean square deviation (MSD) (Equation (15)), and the chi-square (χ^2^) (Equation (16)) were determined [28,29].

(14) $$R^{2}=\frac{\sum_{i=1}^{N}{\left[\left({\mathrm{MR}}_{\exp,i}-{\bar{\mathrm{MR}}}_{\exp,\;i}\right)\left({\mathrm{MR}}_{\mathrm{pred},\;i}-{\bar{\mathrm{MR}}}_{\mathrm{pred},\;i}\right)\right]}^{2}}{\sum_{i=1}^{N}{\left({\mathrm{MR}}_{\exp,i}-{\bar{\mathrm{MR}}}_{\exp,\;i}\right)}^{2}\sum_{i=1}^{N}{\left({\mathrm{MR}}_{\mathrm{pred},\;i}-{\bar{\mathrm{MR}}}_{\mathrm{pred},\;i}\right)}^{2}},$$

(15) $$\mathrm{MSD}=\;{\left[\frac{1}{N}\;\overset{N}{\underset{i=1}{\sum }}{{(\mathrm{MR}}_{\mathrm{pred},\;i}-{\mathrm{MR}}_{\exp,\;i})}^{2}\right]}^{\frac{1}{2}},$$

(16) $$\chi^{2\;}=\frac{1}{N\;-\;n}\;\overset{N}{\underset{i=1}{\sum }}{\left({\mathrm{MR}}_{\mathrm{pred},\;i}-{\;\mathrm{MR}}_{\exp,\;i}\right)}^{2},$$

where ${\mathrm{MR}}_{\exp,i}$ is the experimental moisture content ratio; ${\bar{\mathrm{MR}}}_{\exp,\;i}$ is the mean of the experimental moisture content ratio; ${\mathrm{MR}}_{\exp,i}$ is the moisture content ratio predicted by the model; ${\bar{\mathrm{MR}}}_{\mathrm{pred},\;i}$ is the mean of the moisture content ratio predicted by the model; N is the number of experimental points; n is the number of constants of the model.

#### 2.2.2. Effective Diffusivity

The effective diffusivity indicates the ability to remove water contained in the material and varies according to the temperature conditions and drying air velocity. For its determination, the diffusion theory is used, Fick’s law, which expresses the mass flow per unit area proportional to the water concentration gradient [30]. The effective diffusivities were determined by fitting the net diffusion model (Equation (17)) to the drying experimental data, with an approximation of 10 terms (n = 10). This equation is the analytical solution for Fick’s second law, considering the geometric shape of the samples as approximate to that of an infinite flat plate, both for the peels and seeds, not taking into consideration the volumetric contraction during the process [31].

(17) $$\mathrm{MR}=\frac{X\;-{\;X}_{e}}{X_{0}-{\;X}_{e}}=\frac{8}{\pi^{2}}\sum_{n=0}^{10}\frac{1}{{\left(2n+1\right)}^{2}}\exp\left[-{{\left(2n+1\right)}^{2}\pi^{2}D}_{\mathrm{ef}}\frac{1}{4L^{2}}\right],$$

where D_ef_—effective diffusion coefficient (m^2^/s); n—number of terms in the equation; L—characteristic dimension (half thickness of the sample) (m); t—time (s).

In order to evaluate the effect of the temperature of the drying process on the effective diffusivity of the samples, an Arrhenius equation was used (Equation (18)). Activation energies were determined through the slope of the Ln curve (D_ef_) as a function of the absolute temperature inverse (1/T).

(18) $$D_{\mathrm{ef}}{=D}_{0}\exp\left(\frac{E_{a}}{{\mathrm{RT}}_{a}}\right).$$

where D_0_—Pre-exponential factor (m^2^/s); E_a_—Activation energy (kJ/mol); R—Universal gas constant (8314 kJ/kmol K); T_a_—Absolute temperature (K).

### 2.3. Thermodynamic Properties

The thermodynamic properties of enthalpy (ΔH), entropy (ΔS), and Gibbs free energy (ΔG) were calculated according to Equations (19)–(21), respectively [32,33].

(19) $$∆{H=E}_{a}-\;\mathrm{RT}\;,$$

(20) $$∆S=R\;\left[{\mathrm{In}}_{\left(D0\right)}-{\;\mathrm{In}}^{\;}\left(\begin{array}{c} \;\\ \; \end{array}\frac{K_{b}}{h_{p}}\right)\right]-{\;\mathrm{In}}^{\;}T\;,$$

(21) ∆G=∆H − T∆S,

where ∆H—Enthalpy (J/mol); ∆S—Entropy (J/mol K); ∆G—Gibbs free energy (J/mol); k_b_—Boltzmann’s constant (1.38 × 10^−23^ J/K); h_p_—Planck’s constant (6.626 × 10^−34^ J/s); T—Absolute temperature (K).

### 2.4. Obtaining Flour

After drying, the materials obtained at different temperatures were ground individually in a knife mill (Marconi, model TE 340, Piracicaba, São Paulo, Brazil) to obtain flour.

#### Proximal Composition and Physicochemical Characterization of Pomegranate Peels and Seeds Fresh and Flour

The pomegranate peels and seeds fresh and in flour produced after drying were characterized in triplicate and the results expressed in wet base (wb) in terms of physical-chemical parameters, following the methodologies described in the manual of the AOAC [14]: water content by the g drying greenhouse method at 105 °C until the constant mass; the ash state was provided by incineration in a muffle furnace at 550 °C; proteins using the micro Kjeldahl method, considering the conversion factor for the crude protein of 6.25; the total regards acidity, was determined by titration with 0.1 MN_a_OH; the pH was determined in the crushed samples and diluted in distilled water, being read on a digital potentiometer.

Following the procedures of the AOAC [14], the ascorbic acid content was determined by titration with sodium 2.6 dichlorophenolindophenol (DCFI) until a persistent light pink color was obtained, using oxalic acid as an extractant solution [34]; the total lipid content was determined by the Bligh and Dyer [35] method; and the total carbohydrate content was calculated by difference, subtracting from one hundred the values obtained for water, ash, protein, and lipid content. Results were expressed in g per 100 g of sample (g/100 g).

The quantification of total sugars was determined by the anthrone method, with the glucose standard curve read in a spectrophotometer at 620 nm [36]; the content of reducing sugars followed the procedure proposed by Miller [37], using 3.5-dinitrosalicylic acid (DNS), with the glucose standard curve read in a spectrophotometer at 540 nm; and non-reducing sugars by difference. The crude fiber content of the flours was determined in triplicate, according to the methodology AOAC [14]. The measurement of water activity (aw) was performed in a dew point hygrometer (Aqualab, 3TE model, Decagon Devices, Washington, DC., United States).

The total energy value was calculated considering the conversion factors of the Atwater [38] system, 4 kcal/g for proteins, 9 kcal/g for lipids, and 4 kcal/g for carbohydrates. The results were expressed in kcal per 100 g of sample (kcal/100 g).

## 3. Results and Discussion

### 3.1. Drying Kinetics

Table 2 shows the drying times of pomegranate peel and seed flour at temperatures from 50 to 70 °C.

The higher initial moisture content of the peels resulted in longer drying times than in the seeds drying, so that at the temperature of 70 °C the peels dried at a similar time to the seeds at 50 °C indicating that the structure of the material and the drying temperature are key factors in determining the drying time [39]. Even with longer times, at the end of drying, the peels had a water content between 4.8 and 4.7 times greater than the seed’s water content. Between the drying temperatures of 50 and 70 °C, the drying time was reduced by 14% to 24% in the peels and seeds, respectively. Doymaz [40] and Mphahlele et al. [41] reported considerable increases in drying rates when higher temperatures were used on agro-industrial pomegranate residues. Several other authors report similar results in the drying of grains, fruits, seeds, and agro-industrial residues [42,43,44].

The water content of the pomegranate peel and seed flour varied between 1.26 and 11.84% wb (wet base), with all values within the maximum content for wheat flour recommended by current Brazilian legislation [45], which is 15.00%. The water content close to that of pomegranate peel flour was determined by Farias [46] also for pomegranate peel flour with a water content of 10.56%.

Table 3 and Table 4 present the parameters of the mathematical models adjusted to the drying kinetics experimental data of pomegranate peels and seeds, their respective coefficients of determination (R^2^), mean squared deviations (MSD), and chi-square (χ^2^) at drying temperatures from 50 to 70 °C.

For the two types of flours, all models showed good adjustments to the experimental data presenting R^2^ greater than 0.97. For the pomegranate peel flours (Table 4), the Diffusion Approximation and Verma models presented the best set of R^2^, MSD, and χ^2^. The results presented by Diógenes et al. [47] also attest that the diffusion approximation model presented the best estimates of the drying kinetics curves for all temperatures in all samples of whole pumpkin seeds. Kara and Doymaz et al. [48], when studying the drying of pomegranate residues generated in the juice processing at temperatures of 50, 60, 70, and 80 °C, verified excellent R^2^ and χ^2^ results for the Midilli, Verma, and Diffusion Approximation models for all dehydration temperatures.

From the parameters presented in Table 4, it can be seen that all models resulted in R^2^ greater than 0.98 and that MSD and χ^2^ presented low values. As with pomegranate peel, the Diffusion Approximation model showed the best adjustment coefficients for seeds, standing out at temperatures of 60 and 70 °C, being surpassed only at 50 °C by the modified Henderson and Pabis model. Satisfactory adjustments were also verified by Barros et al. [49] applying mathematical models to experimental data on the drying kinetics of kino (*Cucumis metuliferus*) peels, reporting that the Page and Diffusion Approximation models presented satisfactory adjustments; by Gonçalves et al. [50] for the representation of the green banana pulp drying kinetics at 55, 65 and 75 °C, who also determined that the Diffusion Approximation model stood out concerning the other models tested; by Santos et al. [42] studying a thin layer drying of prickly pear (*Opuntia ficus-indica*) at temperatures of 50, 60 and 70 °C; and by Diógenes et al. [47] studying the drying kinetics of pumpkin seeds at temperatures of 40, 50, 60, 70 and 80 °C also obtaining good fits with the Diffusion Approximation model.

The parameter “k” which represents the constant of the drying rate and reflects the relationship between the effective diffusivity and the diffusion process [51] was checked. It can be used indirectly to evaluate the effect of temperature on the effective diffusivity in the drying period at a decreasing rate, when the net diffusivity controls the process [52]. It increased in most of the models studied, with the addition of the dehydration temperature, indicating that the increase in k implies an increase in the diffusivity of pomegranate peels and seeds when the temperature is increased.

**Figure 1:**
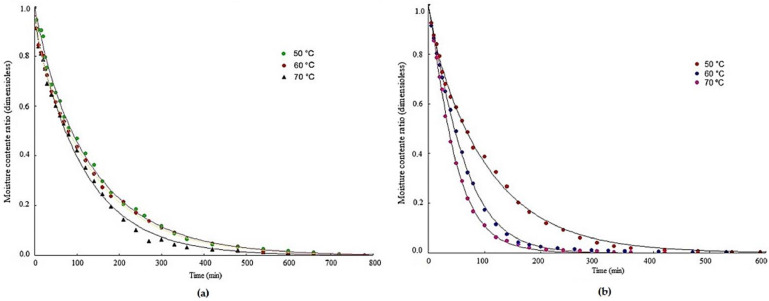
Pomegranate drying kinetics at temperatures of 50, 60 and 70 °C: (**a**) peels and (**b**) seeds.

The drying curves of pomegranate peels and seeds at temperatures of 50, 60, and 70 °C are shown in Figure 1. Different behaviors are observed between the curves of peels (Figure 1a) and seeds (Figure 1b). The seed curve demonstrates a common behavior of agricultural products, presenting a faster loss of water in the initial times, while in the peel curve, the drying rates appear similar over the processing time. In the peels, the temperature differences have less influence on the relative positions of the curves, while in the seeds the temperature curve at 50 °C differs from the others presenting proportionally slower drying and a reduction in the drying time with the temperature increase.

Drying curves similar to those of pomegranate seeds were verified by Santos et al. [42] determining the pomegranate drying kinetics (seed and pulp) at temperatures of 50, 60, and 70 °C, where they reported that the drying curves were influenced by temperature, with a gradual reduction in drying times observed as the drying air temperature was increased; by Moscon et al. [53] analyzing the drying kinetics of quinoa (*Chenopodium quinoa* W.) grains at temperatures of 40, 50, 60, and 70 °C, who also noticed an inverse behavior between temperature and time required for drying, that is, a reduction in drying time and an increase in water removal with increasing temperature; and by Santos et al. [42] who determined the modeling and the pomegranate peel drying kinetics, also verifying the decrease in drying time with increasing temperature. The highest drying rate was obtained at the highest temperature (80 °C) and the time spent to dry the peels at the lowest temperature (50 °C) was almost triple the time required to dry them at the highest temperature. Mphahlele et al. [41] reported in their study of drying kinetics of pomegranate peel (cv. Wonderful) at three temperatures (40, 50, and 60 °C) that the drying rate was higher at the beginning of the process, possibly due to evaporation of the peel surface water, which subsequently decreased with decreasing water content for the entire temperature range, and the drying process occurred mainly in the period at the decreasing rate for the entire temperature range, indicating that the mass transfer occurred by diffusion.

### 3.2. Effective Diffusivity

Table 5 shows the values of effective diffusivities determined from the drying kinetics of pomegranate seeds and peels. Diffusivity is used to indicate the flow of water within a material and is primarily influenced by the material’s water content and temperature.

It is observed that increases in drying temperatures promote increasing values of D_ef_. In addition, the lowest values of this variable were observed in the peels whose adjustments presented better R^2^ for the seeds. There is evidence that the highest D_ef_ seeds were responsible for the greater ease of drying, presenting an increase in temperature and shorter drying times. The increase in D_ef_ is justified because the viscosity of water decreases with increasing temperature, favoring its movement and increasing the diffusion of water in the solid matrix, also affected by the increase in vapor pressure inside the sample [54]. In this way, the water gradient between the sample and the drying air is high, promoting an increase in the effective diffusivity of water [55].

According to Madamba et al. [56], the effective diffusivity values for the drying of plant products are generally of the order from 10^−9^ to 10^−11^ m^2^/s, so the D_ef_ observed for pomegranate in the present study is close to this range, with values for peels varying from 3.7583 × 10^−12^ to 4.6803 × 10^−12^ and for the seeds from 1.3106 × 10^−9^ to 2.5907 × 10^−9^, also close to the range of values observed by other researchers such as Mphahlele et al. [41] who reported effective diffusivities of pomegranate peels at drying temperatures of 40, 50 and 60 °C from 4.05 × 10^−10^, 5.06 × 10^−10^ to 8.10 × 10^−10^ m^2^/s, respectively; Kara and Doymaz. [48] who detected effective diffusivity values between 1.22 × 10^–10^ and 4.29 × 10^–10^ m^2^/s in the pomegranate by-products drying at temperatures from 50 to 80 °C; Kaveh et al. [57] who detected the D_ef_ value of 4.11 × 10^−10^ in the convective drying of pomegranate arils at 50 °C; and by Süfer and Palazoglu [58] who determined D_ef_ values from 3.56 × 10^−11^ to 1.93 × 10^−10^ m^2^/s for dehydrated pomegranate arils at temperatures of 55–75 °C.

Table 6 shows the activation energy values (E_a_) determined from the Ahrrenius-type equation for the effective diffusivities of the peels and seeds.

A value almost three times higher for the E_a_ of the seeds concerning the peels is observed, and the coefficients of determination (R^2^) were greater than 0.96, representing satisfactory adjustments. The result obtained for the seeds is similar to that of Kara and Doymaz et al. [48] that present an E_a_ of 39.66 kJ/mol for the kinetics pomegranate by-products juice; and the result obtained for the peels was lower than 21.98 kJ/mol, as reported by Mphahlele et al. [41].

According to Zhang et al. [59], activation energy reflects the binding capacity of water to materials, indicating the energy required for water molecules to change from a normal state to an active state that is prone to dehydration. Because of this statement, it is observed that pomegranate seeds need more energy for drying to occur. This higher E_a_ for the seeds is related to their physicochemical composition, which in turn presented a much higher fiber content, probably hindering water diffusion and requiring more energy to initiate the process. It is observed that the values of D_ef0_ for pomegranate peels and seeds were close.

### 3.3. Thermodynamic Properties

Table 7 shows the enthalpy, entropy, and Gibbs free energy for drying pomegranate peels and seeds. The seeds surpass the peels in the values of all properties, highlighting greater differences between the enthalpy results.

The enthalpy values (∆H) were inversely proportional to the temperatures, with reduction as the temperature increased. Enthalpy reductions indicate that a smaller amount of energy is required for drying at higher temperatures. According to Araújo et al. [32], considering the product as a thermodynamic system, this behavior occurs due to the partial pressure of water vapor increasing inside the samples while the partial pressure of the air remains constant, thus increasing the surface diffusivity.

Enthalpy is defined as a state function and depends only on the predominant equilibrium state identified by the internal energy, pressure, and volume and under these conditions, it represents the heat absorbed (or released) by the material through external heat transfer (drying) [60]. For both pomegranate samples, the ΔH values obtained were positive, indicating that the drying process was endothermic. Similar behavior was verified by Ghibate et al. [61] evaluating the thermodynamic parameters of drying pomegranate peels who also detected a positive value of ΔH (11.12 kJ/mol) characterizing the endothermic nature of the drying process. Regarding the enthalpy differences between seeds and peels deriving from the distinct material composition and structure, the same conclusion was reached by Koukouch et al. [62] evaluating the whole and defatted olive pomace drying, and by Alves et al. [63] studying the drying of whole and partially defatted baru almonds at 60, 70, and 80 °C.

Entropy (∆S) behaved similarly to enthalpy, having reduced values with increasing temperature. With the increase in the temperature of the drying air and consequent increase in the partial pressure of water vapor in the product, there is also an increase in the excitation of water molecules and a reduction in viscosity, factors that, combined, provide an increase in the speed of the water diffusion process and reduction in entropy in the process [51]. According to Moreira et al. [64], the negative values of entropy are attributed to the existence of chemical adsorption and/or structural modifications of the adsorbent. Araújo et al. [32] also detected negative entropy values in the peanut drying process, with values from −0.1678 to −0.1686 kJ/mol K at temperatures from 40 to 60 °C.

The Gibbs free energy (ΔG) can characterize the drying process as spontaneous or non-spontaneous and indicates the amount of water bound to the product [65]. ΔG values greater than zero (positive) result from the non-spontaneity of processes (at constant pressure and temperature) that require energy input (endergonic processes). Several studies were carried out on the thermodynamic properties of agricultural products and the results for the drying of pomegranate seeds and peels are similar to those of Santos et al. [66] who also detected an ΔG increase in the drying of acuri slices with increasing temperature from 60 to 90 °C, with values ranging from 139.49 to 150.72 kJ/mol, respectively. The results were above to those of Araújo et al. [32] who detected ΔG in peanut drying values between 85.18 and 90.23 kJ/mol for temperatures from 40 to 70 °C. Positive values are expected, since desorption is a non-spontaneous process, as observed in the present study.

### 3.4. Proximal Composition and Physicochemical Parameters of Fresh Pomegranate Peels and Seeds and in Their Flours

Table 8 presents the results of the physicochemical characterization of pomegranate peels and seeds fresh and in the flours obtained from drying at temperatures of 50, 60, and 70 °C.

The water contents of the pomegranate peel flours were statistically different and higher than the contents of the seeds in the three drying conditions, being inferior in all conditions in relation to the fresh samples, which was already expected, since the samples were subjected to dehydration to obtain the flour. In addition, the contents showed a tendency to decrease with increasing drying temperature. The water content of fresh pomegranate peel of 70.74% is close to those determined by Abid et al. [67] evaluating pomegranate peels from different Tunisian ecotypes (‘Acide’, ‘Gabsi’, ‘Nebli’ and ‘Tounsi’), in which the water contents ranged from 67.26 to 73.23%. Regarding the flour close values, they were determined by Hesham et al. [68] who reported in their work a water content of 10.32% for pomegranate peel flour; and by Farias [46] who considers pomegranate peel flour as a low-moisture product, with a water content of 10.56%.

The average value of lipids in fresh pomegranate peels was 0.98%, and in peel flours it ranged from 0.54 to 0.61%, rendering it a product with low lipid content (<3%), according to Brasil [42]. A value of lipids close to that of pomegranate peel flour was determined by Sharifi et al. [69] in pomegranate peel flour dried at 60 °C with a content of 0.67%. Lower values were reported by Santos et al. [70] when studying the physicochemical and biochemical characteristics and proximate composition of pomegranate cv. verifying levels of 0.19% for the peel and 0.20% for the pulp fresh, while for pomegranate seeds fresh and in their flours, the levels of lipids observed were higher than 3%, showing that they are sources of lipids. It is verified that there was a significant increase in the lipid content of the seed flours with the increase in the drying temperature, indicating a concentration of this nutrient due to the drying process. Increased lipid content after processing using heat was reported by Jan et al. [71] during the drying of Nigella seeds, in which they observed contents of 21.00% in the seed fresh and 22.00% after microwave drying. Pomegranate seeds are recognized as a source of lipids, which can result in oil extraction yields ranging approximately from 7 to 27% [72]. The oil extracted from pomegranate seeds, according to Paul and Radhakrishnan [73], can be considered a functional food with bioactive properties, which promotes the prevention of metabolic disorders, and a source of conjugated fatty acids, mainly punicic acid.

Pomegranate seeds and their flours also showed significantly higher protein contents than the peels, and both types of flour showed a tendency to concentrate the protein content with increasing drying temperature. In general, husk flour can be considered a source of protein (minimum of 6%), and the ones with seeds with a high content (minimum of 12%) according to Brasil [42]. The protein content of pomegranate peel flour was close to that observed by Omer et al. [74] who mentioned an average value of 6.52% in sun-dried pomegranate peel; and by Rowayshed et al. [75] who verified protein contents of 13.66% bs and 3.10% bs, respectively, for pomegranate (*Punica granatum* L.) seed and peel flours dehydrated in a drying greenhouse with forced air circulation at 60 ± 5 °C.

The amount of fiber observed in this study confirms the potential for using pomegranate peels and seeds and their flours as food ingredients with a high fiber content (above 6%). It is observed that the contents of pomegranate seeds and their flours were approximately twice those of the peels and their flours. These values were higher than those found by Martínez et al. [76] in six pomegranate cultivars produced in Morocco, obtaining crude fiber contents from 0.9 to 2.1% fresh pomegranate seeds; by Hernándes et al. [77] evaluating the quality of pomegranates (aryls and seeds) grown in southwestern Spain, where crude fiber content was observed in fresh seeds ranging from 1.8 to 2.4%; by Omer et al. [74] investigating dehydrated pomegranate peels (*Punica granatum* L.) with a crude fiber content of 10.50%; and by Farias [46] evaluating the crude fiber content of 7.73% in pomegranate peel flour (*Punica granatum* L.).

The ash content between the pomegranate peels and seeds and between their flours differed statistically, presenting the highest contents by the pomegranate peels and their flours. The ash values obtained in the bark samples were lower than those determined by Kushwaha et al. [78] for pomegranate peel flour dehydrated at 60 °C/18 h with a content of 5.49%, and close to that presented by Omer et al. [74] of 3.43% for pomegranate peel flour. For pomegranate seeds, higher values were quantified by Campos et al. [79] who verified ash contents of 3.13, 4.39, and 2.56% for the fresh seeds of the cultivars Acco, Big Full, and Wonderful; and content close to that was reported by Jalal et al. [80] for pomegranate seed flour, dehydrated in a drying greenhouse with air circulation at 60 ± 5 °C for 6 h, with an ash content of 1.46%.

The carbohydrates present in the pomegranate peel flour were statistically higher than those in the seeds, with both types of flour showing a high content of carbohydrates, with levels close to that of corn flour, 79.1% [81], which is a food rich in carbohydrates. It is observed that these values were similar to those determined by Ismail et al. [82] for pomegranate peel flour with a carbohydrate content of 78.67% and close to that quantified by Ranjitha et al. [83] who detected a value of 66.51% for pomegranate peel flour, dehydrated in a tray dryer at 65 °C for 10 h.

The energy value of the fresh seeds and their flours was higher than that of the fresh peels and their flours, and with the drying process, there was a significant increase in the energy value with the temperature increase. Values above 40 kcal/100 g can be indicative of high energy value foods [45]. A similar value was detected by Nogueira et al. [84] for pomegranate seed flour with a content of 419.52 kcal/100 g. The high energy value of pomegranate seeds is known due to their high lipid content.

The water activity of all samples followed the behavior of the water content, with the peel showing higher values both in the fresh sample and in the flours, decreasing with the drying temperature increase, but without causing statistical differences. The a_w_ of 0.987 of the fresh bark is similar to that reported by Marchi [72], who detected a value of 0.977 in pomegranate peels. After drying, the samples reached an adequate value for conservation because of the limited value of water activity for any microbial development of 0.60 [85], verifying that the application of convective drying is capable of reducing the water content and water activity of the product, making possible the efficient conservation and storage of the studied materials.

The acidity of the fresh peels exceeds that of the seeds by more than 12 times; the difference is reduced with drying, but it remains still greater than 9 times at the three temperatures. Acidity plays a key role in the taste of food [86]. The high acidity of pomegranate peel flour indicates a good ability to flavor infusions, one of the ways in which it is used. Fresh pomegranate seeds and their flours presented acidity below 1%, these values being close to those determined by Ataíde et al. [87] for the fresh pomegranate seeds with values from 0.41 to 0.72% of citric acid and higher than those of pomegranate peels with values ranging from 2.72 to 5.04% of citric acid.

Following the opposite behavior of acidity, the peels presented lower pH values, reaching the lowest values in the flours dried at 50 °C. Peel flours, with values between 3.70 and 3.82, had a pH lower than those determined by Oliveira et al. [88], whose result for pomegranate peel flours was 4.83, while for the seed, the authors obtained a value of 4.55, more acidic than in the seeds flours evaluated in this work.

The results for the contents of total sugars and non-reducing sugars in the peel flour samples were higher than those of the seed flours, while in the fresh samples the behavior was inverse, indicating a high concentration of these compounds in the peel flours. As for the reducing sugars, small amounts were observed, with no significant difference between the samples. Higher content was detected by Santos et al. [70] for the fresh pomegranate peels grown in Oeste Paulista with a value of 6.18% of reducing sugars. Values close to total sugars were detected by Tozzi et al. [89] in freeze-dried pomegranate peel flour, which showed a total sugar concentration of 38.9 g/100 g.

It can be observed from the evaluated results that the composition of the material and the physicochemical characteristics varied significantly between the pomegranate peels and seeds, and that a concentration in most of the constituents occurred in the flours, which can be explained by the removal of part of the water present in the food provided by the drying. The differences between the results obtained and the works in the literature can be influenced by several factors, such as the characteristics of the cultivation system, the cultivated variety, the region, the climate, soil, in addition to the type of drying process and dryer operating conditions [90,91].

## 4. Conclusions

Temperature increments promoted reductions in drying times and equilibrium water content of the studied materials. Among the mathematical models tested, the Diffusion Approximation and Verma model was the one that best fitted the experimental data of pomegranate peel kinetics and the Diffusion Approximation and Modified Henderson and Pabis for the experimental seed data, in all evaluated conditions, presenting the highest values of R^2^, lowest DQM, and χ^2^.

The thermodynamic properties in both drying processes pointed to a non-spontaneous process, with positive values of enthalpy and Gibbs free energy, and negative values of entropy. Enthalpy and entropy values decreased with increasing drying temperature, while Gibbs free energy values increased in the temperature range evaluated in both materials.

The seeds have less water in the composition, dry in shorter times, and reach lower water contents than the peels when submitted to drying at temperatures of 50, 60, and 70 °C.

Pomegranate peel and seed flours have proximal composition and distinct physicochemical characteristics, presenting high fiber, carbohydrate, and energy content. In addition, the peel flours are noteworthy for their mineral content and the seed flours are for their lipid and protein content.

Therefore, the processing and use of pomegranate residues stand out as an excellent technological alternative, since the obtained flours presented important constituents that can be applied in the food and pharmaceutical industry for the enrichment and elaboration of new products.

## Figures and Tables

**Table 1:** Mathematical models adjusted to drying kinetics data.

Model	Equation	Equation	Reference
Diffusion Approximation	MR=a.exp(-k.t)+(1 − a). exp(-k.b.t)	(2)	[16]
Two terms	$\mathrm{MR}=a.\exp(-k.t)+b{.\exp(-k}_{1}.t)$	(3)	[17]
Two-term exponential	MR=a.exp(-k.t)+(1 − t).exp(-k.a.t)	(4)	[18]
Henderson and Pabis Modified	$\mathrm{MR}=a.\exp\left({-k}_{0}.t\right)+b.\exp\left({-k}_{1.t}\right)+c{.\;\exp(-k}_{2.t})$	(5)	[19]
Henderson and Pabis	MR=a.exp(-k.t)	(6)	[20]
Logarithmic	MR=a.exp(-k t)+c	(7)	[21]
Logistic	${\mathrm{MR}=a}_{0}/(1.\mathrm{aexp}\left(k.t\right))$	(8)	[22]
c	MR=exp(-kt)	(9)	[23]
Page	$\mathrm{MR}=\exp(-k{.t}^{n})$	(10)	[24]
Verma	$\mathrm{MR}=a\;.\exp\left(-k\;{.\;t}^{1}\right)+\left(1\;-\;a\right)\;.\exp(-k1.t)$	(11)	[25]
Thompson	${\mathrm{MR}=\exp(-a\;(a}^{2\;}+4.b{.t)}^{0.5})/2.b)$	(12)	[26]
Midilli	$\mathrm{MR}=a.\exp\left(-k{.t}^{n}\right)+b.t$	(13)	[27]

MR—ratio of product water content (dimensionless); t—drying time (min); k, k_0_ and k_1_—drying constants (1/min); a, b, c, n—model coefficients.

**Table 2:** Average values of drying times and water contents of pomegranate peel and seed flour at drying temperatures of 50, 60, and 70 °C.

Pomegranate Sample	Drying Temperature (°C)	Drying Time (min)	Water Content (% bu)	Water Content (% bs)
Peels	50	840	11.84	13.44
	60	780	8.30	9.09
	70	720	7.14	7.70
Seeds	50	710	2.42	2.48
	60	650	1.64	1.67
	70	540	1.26	1.28

**Table 3:** Parameters of the models adjusted to the drying data of pomegranate peel flour at temperatures of 50, 60 and 70 °C.

Model	T (°C)	Parameters						R^2^	MSD	χ^2^
		a	k	b						
Diffusion Approximation	50	0.1043	0.0304	0.2254	-	-	-	0.9984	0.0134	0.0002
	60	0.1185	0.1174	0.0601	-	-	-	0.9994	0.0077	0.0001
	70	0.0781	0.2629	0.0321	-	-	-	0.9982	0.0138	0.0002
Model	T (°C)	a	k_0_	b	k_1_			R^2^	MSD	χ^2^
Two Terms	50	0.8978	0.0069	0.1068	0.0327	-	-	0.9985	0.0133	0.0002
	60	0.1159	0.1089	0.8798	0.0070	-	-	0.9994	0.0076	0.0001
	70	0.5535	0.0087	0.3905	0.0087			0.9968	0.0185	0.0004
Model	T (°C)	a	k					R^2^	MSD	χ^2^
Exponential	50	0.0363	0.2018	-	-	-	-	0.9978	0.0158	0.0003
	60	0.1321	0.0535		-	-	-	0.9986	0.0122	0.0002
	70	0.0793	0.1071	-	-	-	-	0.9977	0.0156	0.0002
Model	T (°C)	a	k					R^2^	MSD	χ^2^
Henderson and Pabis	50	0.9776	0.0074	-	-	-	-	0.9975	0.0167	0.0003
	60	0.9273	0.0075	-	-	-	-	0.9966	0.0187	0.0004
	70	0.9439	0.0087	-	-	-	-	0.9968	0.0185	0.0004
Model	T (°C)	a	k_0_	b	k_1_	c	k_2_	R^2^	MSD	χ^2^
Modified Henderson and Pabis	50	0.1068	0.0327	0.7618	0.0068	0.1359	0.0068	0.9981	0.0133	0.0002
	60	0.0858	0.0348	0.8481	0.0068	0.0666	0.2907	0.9995	0.0066	0.0001
	70	0.3144	0.0087	0.3144	0.0087	0.3149	0.0087	0.9968	0.0185	0.0004
Model	T (°C)	a	k	c				R^2^	MSD	χ^2^
Logarithmic	50	0.9729	0.0076	0.0069	-	-	-	0.9977	0.0163	0.0003
	60	0.9205	0.0078	0.0102	-	-	-	0.9968	0.0181	0.0004
	70	0.9466	0.0086	0.0038				0.9968	0.0184	0.0004
Model	T (°C)	a_0_	a	k				R^2^	MSD	χ^2^
Logistic	50	0.0538	0.0550	0.0075	-	-	-	0.9976	0.0167	0.0003
	60	0.1635	0.1763	0.0076	-	-	-	0.9966	0.0209	0.0004
	70	0.1475	0.1562	0.0087				0.9968	0.0185	0.0004
Model	T (°C)	k						R^2^	MSD	χ^2^
Newton	50	0.0077	-	-	-	-	-	0.9969	0.0189	0.0004
	60	0.0085	-	-	-	-	-	0.9883	0.0344	0.0012
	70	0.0095				-	-	0.9923	0.0288	0.0009
Model	T (°C)	k	n					R^2^	MSD	χ^2^
Page	50	0.0110	0.9256	-	-	-	-	0.9981	0.0139	0.0002
	60	0.0194	0.8245	-	-	-	-	0.9985	0.0121	0.0002
	70	0.0160	0.8868				-	0.9958	0.0210	0.0005
Model	T (°C)	a	k	k_1_				R^2^	MSD	χ^2^
Verma	50	0.1042	0.0304	0.0068	-	-	-	0.9984	0.0134	0.0002
	60	0.1184	0.1174	0.0070	-	-	-	0.9994	0.0077	0.0001
	70	0.0780	0.2633	0.0084				0.9982	0.0138	0.0002
Model	T (°C)	a	b					R^2^	MSD	χ^2^
Thompson	50	−11.741	0.0946	-	-	-		0.9980	0.0150	0.0002
	60	−3.8820	0.0415	-	-	-		0.9958	0.0208	0.0005
	70	−10.385	0.1037					0.9935	0.0263	0.0008
Model	T (°C)	a	k	n	b			R^2^	MSD	χ^2^
Midilli	50	1.0053	0.0118	0.9118	0.000007	-	-	0.9983	0.0138	0.0002
	60	0.9803	0.0177	0.8354	0.000022	-	-	0.9992	0.0090	0.0001
	70	0.9586	0.0113	0.9480	0.000013			0.9970	0.0178	0.0004

k-drying constants; a, b, c, k_0_, k_1_, k_2_, n-coefficients of the models.

**Table 4:** Model parameters adjusted to drying data of pomegranate seed flour at temperatures of 50, 60, and 70 °C.

Model	T (°C)	Parameters						R^2^	MSD	χ^2^
		a	k	b						
Diffusion Approximation	50	0.0725	0.0891	0.1054				0.9987	0.0118	0.0002
	60	0.0070	0.0251	0.9932				0.9995	0.0103	0.0001
	70	0.2045	0.0836	0.2878				0.9995	0.0074	0.0001
Model	T (°C)	a	k_0_	b	k_1_			R^2^	MSD	χ^2^
Two Terms	50	0.0729	0.0896	0.9276	0.0094	-	-	0.9987	0.0041	0.0002
	60	0.5113	0.0161	0.5113	0.0161	-	-	0.9967	0.0195	0.0045
	70	0.5208	0.0211	0.5207	0.0211	-	-	0.9976	0.0152	0.0003
Model	T (°C)	a	k					R^2^	MSD	χ^2^
Exponential	50	0.0633	0.1497	-	-	-	-	0.9986	0.0123	0.0002
	60	0.0033	4.7941	-	-	-	-	0.9960	0.0214	0.0001
	70	0.0026	7.5399	-	-	-	-	0.9958	0.0216	0.0005
Model	T (°C)	a	k					R^2^	MSD	χ^2^
Henderson and Pabis	50	0.9655	0.0098	-	-	-	-	0.9979	0.0152	0.0002
	60	1.0230	0.0161	-	-	-	-	0.9967	0.0195	0.0004
	70	1.0415	0.0211	-	-	-	-	0.9976	0.0163	0.0003
Model	T (°C)	a	k_0_	b	k_1_	c	k_2_	R^2^	MSD	χ^2^
Modified Henderson and Pabis	50	−0.3878	0.0050	1.2822	0.0075	0.1072	0.0690	0.9992	0.0101	0.0001
	60	0.3410	0.0161	0.3410	0.0161	0.3410	0.0161	0.9967	0.0196	0.0005
	70	0.3543	0.0174	0.3543	0.0174	0.3543	0.0174	0.9976	0.0164	0.0003
Model	T (°C)	a	k	c				R^2^	MSD	χ^2^
Logarithmic	50	0.9672	0.0098	−0.0026	-	-	-	0.9979	0.0151	0.0003
	60	1.0290	0.0158	−0.0086	-	-	-	0.9963	0.0188	0.0004
	70	1.0436	0.0209	0.0031	-	-	-	0.9976	0.0162	0.0003
Model	T (°C)	a_0_	a	k				R^2^	MSD	χ^2^
Logistic	50	0.1596	0.1653	0.0099	-	-	-	0.9979	0.0152	0.0002
	60	0.1263	0.1235	0.0161	-	-	-	0.9967	0.0195	0.0004
	70	0.1038	0.0997	0.0211	-	-	-	0.9976	0.0163	0.0003
Model	T (°C)	k						R^2^	MSD	χ^2^
Newton	50	0.0104	-	-	-	-	-	0.9964	0.0201	0.0004
	60	0.0157	-	-	-	-	-	0.9962	0.0211	0.0005
	70	0.0200	-	-	-	-	-	0.9960	0.021	0.0005
Model	T (°C)	k	n					R^2^	MSD	χ^2^
Page	50	0.0151	0.9171	-	-	-	-	0.9981	0.0142	0.0002
	60	0.0092	1.1290	-	-	-	-	0.9987	0.0122	0.0002
	70	0.0112	1.1523	-	-	-	-	0.9993	0.0086	0.0001
Model	T (°C)	a	k	k_1_				R^2^	MSD	χ^2^
Verma	50	0.0725	0.0891	0.0094	-	-	-	0.9987	0.0118	0.0002
	60	−6.5085	0.0260	0.0242	-	-	-	0.9991	0.0103	0.0001
	70	0.0422	0.0200	0.0200	-	-	-	0.9960	0.0211	0.0005
Model	T (°C)	a	b					R^2^	MSD	χ^2^
Thompson	50	−13.716	0.1429	-	-	-	-	0.9972	0.0176	0.0003
	60	−2445.7	1.9007	-	-	-	-	0.9962	0.0211	0.0005
	70	−2054.4	1.9073	-	-	-	-	0.9960	0.0211	0.0005
Model	T (°C)	a	k	n	b			R^2^	MSD	χ^2^
Midilli	50	0.9884	0.0142	0.9248	0.0000	-	-	0.9984	0.0131	0.0002
	60	0.9723	0.0068	1.1928	0.0000	-	-	0.9981	0.0101	0.0001
	70	1.0011	0.0111	1.1537	0.0000	-	-	0.9996	0.0082	0.0001

k-drying constants; a, b, c, k_0_, k_1_, k_2_, n-coefficients of the models.

**Table 5:** Effective diffusivities (D_ef_.) were obtained in the drying kinetics of pomegranate peels and seeds at temperatures of 50, 60, and 70 °C.

Pomegranate	T (°C)	D_ef_ (m^2^/s)	R^2^
Peels	50	3.7583 × 10^−12^	0.9829
	60	4.1243 × 10^−12^	0.9957
	70	4.6803 × 10^−12^	0.9901
Seeds	50	1.3106 × 10^−9^	0.9874
	60	2.0267 × 10^−9^	0.9690
	70	2.5907 × 10^−9^	0.9667

**Table 6:** The activation energy (E_a_),coefficients of determination (R^2^) and pre exponential factor (D_ef0_) of pomegranate peels and seeds.

Pomegranate	D_ef0_	E_a_ (kJ/mol)	R^2^
Peels	1.599 × 10^−5^	10.60	0.9889
Seeds	1.659 × 10^−5^	31.39	0.9798

**Table 7:** Thermodynamic properties of drying kinetics of dried pomegranate peels and seeds at temperatures of 50, 60, and 70 °C.

Pomegranate	T (°C)	ΔH (kJ/mol)	ΔS (kJ/mol K)	ΔG (kJ/mol)
Peels	50	7.9133	−0.43311	147.8717
	60	7.8302	−0.43306	152.2040
	70	7.7471	−0.43301	156.5389
Seeds	50	28.7033	−0.41562	163.0110
	60	28.6202	−0.41225	165.9611
	70	28.5371	−0.41045	169.3844

**Table 8:** Proximal composition and physicochemical characterization of fresh pomegranate peels and seeds and flours in wet base obtained after drying at temperatures of 50, 60, and 70 °C.

Parameter (bu)	Freshsample		Flours		
			50 °C	60 °C	70 °C
Water content (%)	Peels	70.74 ± 0.06 aA	11.41 ± 0.16 aB	10.41 ± 0.15 aC	9.42 ± 0.06 aD
	Seeds	36.60 ± 0.09 bA	4.71 ± 0.10 bB	4.06 ± 0.04 bC	3.73 ± 0.12 bC
Lipids (%)	Peels	0.98 ± 0.01 bB	0.54 ± 0.01 bA	0.61 ± 0.01 bA	0.60 ± 0.01 bA
	Seeds	3.14 ± 0.02 aD	8.43 ± 0.05 aC	9.28 ± 0.04 aB	10.62 ± 0.15 aA
Proteins (%)	Peels	3.40 ± 0.09 bC	5.45 ± 0.30 bB	6.50 ± 0.10 bA	6.64 ± 0.27 bA
	Seeds	7.38 ± 0.04 aD	10.61 ± 0.37 aC	12.21 ± 0.24 aB	13.64 ± 0.29 aA
Fibers (%)	Peels	11.00 ± 0.37 bD	21.06 ± 0.03 bC	22.60 ± 1.54 bB	26.19 ± 0.39 bA
	Seeds	20.85 ± 0.09 aD	41.31 ± 0.27 aC	43.81 ± 0.14 aB	45.94 ± 0.25 aA
Ashes (%)	Peels	1.03 ± 0.02 aC	3.73 ± 0.05 aA	3.72 ± 0.08 aA	3.53 ± 0.06 aB
	Seeds	0.86 ± 0.01 bC	1.48 ± 0.03 bB	1.59 ± 0.01 bA	1.55 ± 0.04 bA
Carbohydrates (%)	Peels	24.74 ± 0.38 bC	78.90 ± 0.41 aB	78.76 ± 0.51 aB	79.81 ± 0.42 aA
	Seeds	51.99 ± 0.33 aD	73.21 ± 0.55 bA	71.49 ± 0.38 bB	68.81 ± 0.56 bC
Energetic value (kcal/100 g)	Peels	113.30 ± 0.14 bD	342.27 ± 0.18 bC	346.53 ± 0.15 bB	351.18 ± 0.11 bA
	Seeds	265.86 ± 0.08 aD	411.14 ± 0.06 aC	418.37 ± 0.09 aB	425.97 ± 0.20 aA
Water activity (a_w_)	Peels	0.987 ± 0.001 aA	0.380 ± 0.001 aB	0.369 ± 0.001 aC	0.362 ± 0.001 aD
	Seeds	0.972 ± 0.001 bA	0.244 ± 0.001 bB	0.235 ± 0.002 bC	0.228 ± 0.001 bD
Total titratable acidity (%citric acid)	Peels	1.52 ± 0.03 aC	6.96 ± 0.04 aA	6.87 ± 0.04 aAB	6.79 ± 0.07 aB
	Seeds	0.13 ± 0.01 bB	0.73 ± 0.04 bA	0.73 ± 0.08 bA	0.75 ± 0.07 bA
pH	Peels	4.07 ± 0.01 bA	3.70 ± 0.01 bD	3.77 ± 0.01 bC	3.82 ± 0.01 bB
	Seeds	5.45 ± 0.01 aA	4.97 ± 0.01 aD	5.17 ± 0.01 aC	5.28 ± 0.01 aB
Total sugars (% glucose)	Peels	1.81 ± 0.004 bD	16.80 ± 0.03 aA	15.70 ± 0.04 aB	12.72 ± 0.03 aC
	Seeds	3.22 ± 0.004 aD	6.24 ± 0.02 bA	4.18 ± 0.01 bC	4.68 ± 0.05 bB
Reducing sugars (% glucose)	Peels	0.22 ± 0.004	0.15 ± 0.002	0.14 ± 0.004	0.13 ± 0.002
	Seeds	0.21 ± 0.001	0.08 ± 0.002	0.05 ± 0.002	0.05 ± 0.002
Non-reducing sugars (% sucrose)	Peels	1.50 ± 0.01 bD	15.81 ± 0.04 aA	14.77 ± 0.05 aB	11.95 ± 0.04 aC
	Seeds	2.82 ± 0.001 aD	5.85 ± 0.02 bA	3.91 ± 0.01 bC	4.39 ± 0.05 bB

Means followed by the same lowercase letters in columns and uppercase in rows do not differ statistically by Tukey’s test at 5% probability.

## Data Availability

Data is contained within the article.
